# Correlation Between Ultrasonographic Placental Thickness and Adverse Fetal and Neonatal Outcomes

**DOI:** 10.7759/cureus.56410

**Published:** 2024-03-18

**Authors:** Seema Rawal, Smriti Ray, Neeraj Sharma

**Affiliations:** 1 Obstetrics and Gynaecology, Dr. Baba Saheb Ambedkar Medical College and Hospital, Delhi, IND; 2 Reproductive Medicine, Indira Gandhi Institute of Medical Sciences (IGIMS), Patna, IND

**Keywords:** thick placenta, perinatal outcome, congenital abnormalities, hydrops, low birth weight, fgr, placental thickness, placenta

## Abstract

Introduction

The placenta is often overlooked in the routine evaluation of normal gestations, receiving attention only when abnormalities are detected. Placental thickness can serve as a good predictor of fetal growth and birth weight, especially in the second trimester.In this prospective study, we measured placental thickness in the second and third trimesters of singleton pregnancies and identified an association between placental thickness and adverse outcomes such as congenital anomalies, fetal growth restriction (FGR), prematurity, low birth weight, stillbirth, and hydrops fetalis.

Methodology

A total of 298 patients aged 20 to 33 years with a singleton pregnancy and regular cycles, who were sure of the date of their last menstrual period, were observed. Placental thickness was measured by ultrasound at 18-20 and 30-32 weeks, and patients were divided into three groups. Group A consisted of patients with normal placental thickness. Group B included patients with a thin placenta (below the 10th percentile). Group C consisted of patients with a thick placenta (above the 95th percentile). The correlation between placental thickness and the fetal and neonatal outcome was observed.

Results

Out of 298 patients, 82 (27.5%) were primigravida and 216 (72.4%) were multigravida. At 18-20 weeks, premature birth was observed in one patient (7.69%) in Group C and six patients (20%) in Group B, compared with eight patients (3.14%) in Group A. At 30-32 weeks, premature birth was seen in two patients (16.67%) in Group C and 11 patients (36.67%) in Group B, compared with two patients (0.78%) in Group A. At 18-20 weeks of gestation, low birth weight was observed for three patients (23.08%) in Group C and 16 patients (53.33%) in Group B, compared with 15 patients (5.88%) in Group A. At 30-32 weeks, low birth weight was observed for four patients (33.33%) in Group C and 19 patients (63.33%) in Group B compared with 11 patients (4.30%) in Group A. A significant association was found between a thin placenta and low birth weight and prematurity at both 18-20 and 30-32 weeks of gestation. Two patients (13.33%) had major congenital abnormalities and a thick placenta at 18-20 weeks. In Group C, hydrops were observed in two patients (15.38%) at 18-20 weeks and two patients (16.67%) at 30-32 weeks. A significant association was found between a thick placenta and hydrops. At 30-32 weeks, 13 patients (43.33%) in Group B had developed FGR compared with six patients (2.34%) with a normal placenta. A significant association was found between a thin placenta and FGR. One patient (7.69%) with a thick placenta had a stillbirth, indicating a nonsignificant association.

Conclusions

A positive correlation was observed between congenital anomalies and hydrops and a thick placenta, whereas FGR, preterm labor, prematurity, and low birth weight were associated with a thin placenta. Subnormal placental thickness for a particular gestational age may be the earliest sign of FGR. A sonographically identified abnormal placenta should alert clinicians to the possibility of a compromised perinatal outcome and the need for evaluation and close follow-up.

## Introduction

The placenta is a fetal organ with essential metabolic, endocrine, and immunologic functions. It is responsible for providing nutrition and facilitating respiration and excretion for the fetus. Additionally, it acts as a barrier and protects the fetus from harmful agents [1]. The placenta is often overlooked in routine evaluations of normal gestations, receiving attention only when abnormalities are detected. Although uncommon, the threshold for recognizing placental abnormalities should be low due to the potential risk of maternal and fetal morbidity and mortality.

The incidence and clinical significance of thick placentas are not well-known. However, associations have been observed with conditions such as diabetes mellitus, infections, and hydrops fetalis. Other reported correlations include congenital anomalies and abruptio placentae [2,3]. Placental dysfunction can lead to a thick placenta through compensatory proliferation and edema of the placental villi. This pathological finding does not benefit fetal growth [4,5].

Placentomegaly occurs when the placental thickness at the center exceeds 4 cm in the second trimester and 6 cm in the third trimester [6]. Placental thickness seems to be a promising parameter for estimating gestational age (GA) and predicting fetomaternal outcomes, as it increases along with GA. Placental thickness is also a good predictor of fetal growth and birth weight, especially in the second trimester [7]. Abnormal placental thickness in the second trimester can be an early indicator of diseases and fetal abnormalities. We aim to establish placental thickness as an indicator for detecting at-risk fetuses, allowing for close follow-up and early interventions where necessary.

## Materials and methods

Data collection method

After obtaining ethical and scientific committee clearance, this prospective observational study was conducted for 18 months in the Department of Obstetrics and Gynaecology at Dr. Baba Saheb Ambedkar Medical College and Hospital in New Delhi.

We selected a sample of 300 patients. Patients fulfilling the eligibility criteria were included in the study after giving written consent. All patients aged 21-35 years with singleton pregnancies who were certain of the date of their last menstrual period and had regular menstrual cycles were included. Patients with multifetal pregnancies; irregular menstrual cycles; a low-lying placenta; a history of preeclampsia, diabetes mellitus, hypertension, and epilepsy; or previous cesarean delivery were excluded.

All ultrasound scans were conducted following the Pre-Conception and Pre-Natal Diagnostic Techniques (Prohibition of Sex Selection) (PC-PNDT) Act. Obstetric ultrasounds were performed using a Siemens Acuson X300 machine (Siemens Medical Solutions, Malvern, PA) with a 3.5 MHz curvilinear transducer through the transabdominal route. The placenta was scanned when the patient was in a supine position and had a moderately distended bladder. The transducer was placed on the skin surface after a coupling agent was applied. The transducer was oriented to scan perpendicular to both the chorionic and basal plates. The placenta was identified as a hyperechoic area separated from the fetus by a hypoechoic area of amniotic fluid. At the level of cord insertion, a straight line was drawn up to the maternal surface of the placenta, and the placental thickness was noted in the cross-section.

Placental thickness was measured from the echogenic chorionic plate to the placental myometrial interface near the mid-placental portion. The myometrium and retroplacental veins were excluded from the measurements. All measurements with the uterus are in a relaxed state because the intervillous space fills with blood during contractions and the placental thickness becomes spuriously increased. The placenta was labeled as thin if below the 10th percentile and as thick if above the 95th percentile. Placental thickness was measured at 18-21 weeks when patients presented for a routine anomaly scan. Patients were asked to visit the Antenatal Care (ANC) clinic routinely, as per protocol. The second scan was taken at 30-32 weeks, and placental thickness was measured.

The patients were divided into three groups. Group A consisted of patients with normal placental thickness (between the 10th and 95th percentile). Group B consisted of patients with a thin placenta (below the 10th percentile). Group C included patients with a thick placenta (above the 95th percentile). All groups were monitored until delivery, and Group A, Group B, and Group C were compared with each other. Subsequently, the abnormal placental thickness was analyzed for comparison in terms of adverse fetal and neonatal outcomes among each group.

Statistical analysis

For statistical analysis, categorical variables were presented as numbers and percentages (%). Conversely, quantitative data were presented as means ± standard deviation (SD) and as medians with 25th and 75th percentiles (interquartile range). Data normality was assessed using the Kolmogorov-Smirnov test. Nonparametric tests were utilized in cases where the data were not normally distributed. Non-normally distributed quantitative variables were analyzed using the Mann-Whitney test (for two groups) and the Kruskal-Wallis test (for more than two groups). Qualitative variables were analyzed using the chi-square test. Fisher’s exact test was used for any cell with an expected value of less than five.

Data entry was done in Microsoft Excel, and the final analysis was conducted using IBM SPSS Statistics for Windows, Version 21.0 (IBM Corp., Armonk, NY). A *P*-value of <0.05 was considered statistically significant.

## Results

Among 300 patients under study, 82 (27.33%) were primigravida, while 218 (72.67%) were multigravida. The mean age of the study population was 24.86 ± 2.8 years, with an age range of 20-33 years. Two patients showed major congenital abnormalities; therefore, pregnancy was terminated before 20 weeks of gestation. The remaining 298 patients were scanned at 30-32 weeks, and fetal outcome was observed.

At a GA of 18-20 weeks, the mean placental thickness was 2.07 ± 0.5 cm. Among these 300 study subjects, the placental thickness was labeled as thin when it was below the 10th percentile and thick when it was above the 95th percentile. We set the cutoff value for a thin and thick placenta at 18-20 weeks at 1.49 and 2.975 cm, respectively. At 30-32 weeks, placental thickness was studied in 298 subjects. The mean placental thickness was 3.12 ± 0.4 cm, and 12 patients had a thick placenta. The cutoff value for a thin and thick placenta at 30-32 weeks was set at 2.569 and 3.89 cm, respectively (Tables 1-2). Of the 300 subjects, two patients (13.33%) with a thick placenta were observed to have congenital anomalies. The *P*-value was 0.002, which was significant (Table 3). These patients were offered termination of pregnancy. Fetal growth restriction (FGR) was observed in 11 patients (36.67%) with a thin placenta at 18-20 weeks and 13 patients (43.33%) with a thin placenta at 30-32 weeks. A significant association (*P* ≤ 0.0001) between FGR and a thin placenta (Tables 4-5).

**Table 1:** Distribution of placental thickness (cm; 18-20 weeks). SD, standard deviation

Placental thickness (cm; 18-20 weeks)	Frequency, *n*	Percentage
Thin placenta (<1.49)	30	10.00%
Normal placenta (1.49-2.975)	255	85.00%
Thick placenta (>2.975)	15	5.00%
Mean ± SD	2.07 ± 0.5	
Median (25th-75th percentile)	2 (1.84-2.19)	
Range	1.09-4.47	

**Table 2:** Distribution of placental thickness (cm; 30-32 weeks). SD, standard deviation

Placental thickness (cm; 30-32 weeks)	Frequency, *n*	Percentage
Thin placenta (<2.569)	30	10.07%
Normal placenta (2.569-3.890)	256	85.91%
Thick placenta (>3.89)	12	4.03%
Mean ± SD	3.12 ± 0.4	
Median (25th-75th percentile)	3.1 (2.97-3.27)	
Range	2.04-5.6	

**Table 3:** Association of congenital anomaly with placental thickness (cm; 18-20 weeks). ^*^P-value was determined using Fisher’s exact test.

Congenital anomaly	Thin placenta (<1.49 cm; *n *= 30)	Normal placenta (1.49-2.975 cm; *n *= 255)	Thick placenta (>2.975 cm; *n *= 15)	Total	*P*-value
Absent	30 (100%)	255 (100%)	13 (86.67%)	298 (99.33%)	0.002^*^
Present	0 (0%)	0 (0%)	2 (13.33%)	2 (0.67%)	

**Table 4:** Association of FGR detection with placental thickness(cm; 18-20 weeks). ^*^P-value was determined using Fisher’s exact test. FGR, fetal growth restriction

FGR detection	Thin placenta(<1.49 cm; *n *= 30)	Normal placenta (1.49-2.975 cm; *n *= 255)	Thick placenta (>2.975 cm; *n *= 13)	Total	*P*-value
Absent	19 (63.33%)	247 (96.86%)	13 (100%)	279 (93.62%)	<0.0001^*^
Present	11 (36.67%)	8 (3.14%)	0 (0%)	19 (6.38%)	
Total	30 (100%)	255 (100%)	13 (100%)	298 (100%)	

**Table 5:** Association of FGR detection with placental thickness (cm; 30-32 weeks). ^*^P-value was determined using Fisher’s exact test. FGR, fetal growth restriction

FGR detection	Thin placenta (<2.569 cm; *n *= 30)	Normal placenta (2.569-3.890 cm; *n *= 256)	Thick placenta (>3.89 cm; *n *= 12)	Total	*P*-value
Absent	17 (56.67%)	250 (97.66%)	12 (100%)	279 (93.62%)	<0.0001^*^
Present	13 (43.33%)	6 (2.34%)	0 (0%)	19 (6.38%)	
Total	30 (100%)	256 (100%)	12 (100%)	298 (100%)	

The mean gestational age at birth was 37.44 ± 1.66 weeks in patients with a thin placenta and 37.97 ± 1.64 weeks in patients with a thick placenta at 18-20 weeks. The mean gestational age at birth was 36.85 ± 1.86 weeks in patients with a thin placenta and 37.29 ± 1.44 weeks in patients with a thick placenta at 30-32 weeks. The mean gestational age in patients with normal placental thickness was 38.62 ± 1.26 weeks and 38.72 ± 1.12 weeks at 18-20 and 30-32 weeks, respectively. The mean gestational age at birth was lower in patients with thin placentas at 18-20 and 30-32 weeks.

At 18-20 weeks, the mean birth weight was 2.17 ± 0.54 kg in patients with a thin placenta compared with 2.82 ± 0.45 kg in patients with a thick placenta and 2.77 ± 0.4 kg in patients with a normal placenta. At 30-32 weeks, the mean birth weight was 2.19 ± 0.49 kg in patients with a thin placenta compared with 2.83 ± 0.57 kg in patients with a thick placenta and 2.76 ± 0.41 kg in patients with a normal placenta. The mean birth weight was lower among patients with a thin placenta than for those with normal placental thickness.

Premature birth was seen in six patients (20%) with a thin placenta and 11 patients (36.67%) with a thin placenta at 18-20 weeks and 30-32 weeks, respectively. A significant association was observed between prematurity and a thin placenta (*P* = 0.001). Low birth weight was observed in 16 patients (53.33%) with a thin placenta at 18-20 weeks and in 19 patients (63.33%) at 30-32 weeks. A significant association (*P* ≤ 0.0001) was found between a thin placenta and low birth weight at 18-20 and 30-32 weeks. Hydrops were observed in two patients (15.38%) with a thick placenta at 18-20 and 30-32 weeks. A significant association was found between a thick placenta and hydrops (*P* = 0.002). One patient had a stillbirth, which had a statistically significant correlation with a thick placenta. Tables 6-7 show fetal and neonatal outcomes and their correlation with placental thickness.

**Table 6:** Association between neonate outcomes and placental thickness (cm; 18-20 weeks). ^*^P-value was determined using Fisher’s exact test. ^†^*P*-value was determined using the Kruskal-Wallis test. NICU, neonatal intensive care unit; SD, standard deviation

Neonate outcome	Thin placenta (<1.49 cm)	Normal placenta (1.49-2.975 cm)	Thick placenta (>2.975 cm)	Total	*P*-value
Neonate outcome					
Fetal distress	1 (3.33%)	4 (1.57%)	3 (25%)	8 (2.69%)	0.003^*^
Premature	6 (20%)	8 (3.14%)	1 (7.69%)	15 (5.03%)	0.001^*^
Low birth weight	16 (53.33%)	15 (5.88%)	3 (23.08%)	34 (11.41%)	<0.0001^*^
NICU admission	13 (43.33%)	11 (4.31%)	3 (25%)	27 (9.09%)	<0.0001^*^
Stillbirth	0 (0%)	0 (0%)	1 (7.69%)	1 (0.34%)	0.044^*^
Hydrops	0 (0%)	0 (0%)	2 (15.38%)	2 (0.67%)	0.002^*^
Gestational age at birth (weeks)					
Mean ± SD	37.44 ± 1.66	38.62 ± 1.26	37.97 ± 1.64	38.47 ± 1.37	<0.0001^†^
Median (25th-75th percentile)	37.71 (37.143-38.571)	38.57 (38-39.571)	37.57 (37-38.714)	38.57 (37.714-39.571)	
Range	32.14-39.86	34-40.86	34.43-40.57	32.14-40.86	
Birth weight (kg)					
Mean ± SD	2.17 ± 0.54	2.77 ± 0.4	2.82 ± 0.45	2.71 ± 0.46	<0.0001^†^
Median (25th-75th percentile)	2.16 (1.802-2.495)	2.76 (2.545-2.99)	2.98 (2.45-3.2)	2.76 (2.515-2.99)	
Range	1.1-3.26	1.56-3.92	2.01-3.23	1.1-3.92	
Apgar at 1 minute					
Mean ± SD	7.87 ± 0.94	8.34 ± 0.71	7.33 ± 1.5	8.25 ± 0.81	0.002^†^
Median (25th-75th percentile)	8 (7-8.75)	8 (8-9)	8 (6-8.25)	8 (8-9)	
Range	6-9	5-9	5-9	5-9	

**Table 7:** Association between neonate outcomes and placental thickness (cm; 30-32 weeks). ^*^P-value was determined using Fisher’s exact test. ^†^*P*-value was determined using the Kruskal-Wallis test. NICU, neonatal intensive care unit; SD, standard deviation

Neonate outcome	Thin placenta (<2.569 cm)	Normal placenta (2.569-3.890 cm)	Thick placenta (>3.89 cm)	Total	*P*-value
Neonate outcome					
Fetal distress	2 (6.67%)	3 (1.17%)	3 (27.27%)	8 (2.69%)	0.0003^*^
Premature	11 (36.67%)	2 (0.78%)	2 (16.67%)	15 (5.03%)	<0.0001^*^
Low birth weight	19 (63.33%)	11 (4.30%)	4 (33.33%)	34 (11.41%)	<0.0001^*^
NICU admission	15 (50%)	7 (2.73%)	5 (45.45%)	27 (9.09%)	<0.0001^*^
Stillbirth	0 (0%)	0 (0%)	1 (8.33%)	1 (0.34%)	0.04^*^
Hydrops	0 (0%)	0 (0%)	2 (16.67%)	2 (0.67%)	0.001^*^
Gestational age at birth(weeks)					
Mean ± SD	36.85 ± 1.86	38.72 ± 1.12	37.29 ± 1.44	38.47 ± 1.37	<0.0001^†^
Median (25th-75th percentile)	37.07 (36-37.929)	38.71 (38-39.571)	37.14 (37-38.571)	38.57 (37.714-39.571)	
Range	32.14-40.29	34-40.86	34.43-38.71	32.14-40.86	
Birth weight (kg)					
Mean ± SD	2.19 ± 0.49	2.76 ± 0.41	2.83 ± 0.57	2.71 ± 0.46	<0.0001^†^
Median (25th-75th percentile)	1.92 (1.9-2.51)	2.76 (2.548-2.99)	3 (2.3-3.2)	2.76 (2.515-2.99)	
Range	1.43-3.26	1.1-3.92	2.01-3.87	1.1-3.92	
Apgar at 1 minute					
Mean ± SD	7.8 ± 1.03	8.36 ± 0.68	6.91 ± 1.3	8.25 ± 0.81	<0.0001^†^
Median (25th-75th percentile)	8 (7-9)	8 (8-9)	7 (6-8)	8 (8-9)	
Range	6-9	5-9	5-9	5-9	

## Discussion

The placenta has been the object of ultrasonographic scrutiny for some time. Ultrasonography has been used to characterize placental position and morphology in such entities as immune and nonimmune hydrops fetalis, gestational diabetes mellitus (GDM), and intrauterine growth retardation. Additionally, abnormal placental thickness is well-recognized as a diagnostic predictor of a wide spectrum of pathologic events [8].

Generally, *thick placenta* is a sonographic term. The cutoff value for defining a thick placenta varies based on GA, measurement approaches, and the condition of the mother and the fetus. Several studies have evaluated the applicability of sonographic placental thickness screening in different trimesters and found a positive linear relationship between GA and placental thickness. Regarding published cutoff values for abnormal placental thickness, La Torre et al., Hoddick et al., and Dombrowski et al. stated that placental thickness should not exceed 40 mm at any GA [8-10]. Table 8 shows various studies’ cutoff values for placental thickness at different gestation periods [11-14].

**Table 8:** Several studies’ cutoffs for thick and thin placentas. MOM, multiple of median

Study	Enrollment (*n*)	Placental thickness range (mm)	Gestation period (weeks)	Cutoff to define a thickened placenta
Elchalal et al. [11]	561	27.3 ± 5.2 mm	20-22	35 mm
		38.2 ± 8.4 mm	32-34	>51 mm
Vachon-Marceau et al. [12]	991	11.8-17.8 mm	11-14	1.2 MOM
Di Wan Masliza et al. [13]	113	28.2 ± 7.57 mm	20-22	>30 mm
		41.6 ± 9.26 mm	30-32	>40 mm
Nagpal et al. [14]	130	33.45 ± 1.62 mm	32	>35.7 mm
		35.7 ± 2.08 mm	36	>39.9 mm
Our study	300	2.07 ± 0.5 cm	18-20	29.7 mm
		3.12 ± 0.4 cm	30-32	38.9 mm

In this study, two patients(13.33%) with a thick placenta showed congenital anomalies at a GA of 18-20 weeks compared with no cases in patients with normal placental thickness or a thin placenta, which was statistically significant (*P* = 0.002). Similarly, Miwa et al. observed a congenital anomaly in 9.4% of patients with a thick placenta compared with 3.2% of patients with normal placental thickness [15]. Placental dysfunction could cause increased placental thickness, which requires evaluation.

Suseela et al. observed that Rh isoimmunization, GDM, non-immune hydrops, and fetal anomalies caused a thick placenta in 22 patients, which corresponds to our findings that congenital anomalies and hydrops are associated with a thick placenta [16]. Raio et al. observed that a sonographic thick, heterogeneous appearance of the placenta is strongly associated with intrauterine and neonatal death [17]. In our study, we observed one stillbirth, which was seen to be significantly associated with a thick placenta.

Nagpal et al. observed that neonatal outcomes in terms of birth weight, Apgar score, and neonatal intensive care unit (NICU) admission were better for women with normal placental thickness than those with thick placenta [14]. However, our study showed a statistically significant correlation between NICU admission and low birth weight and a thin placenta at 18-20 and 30-32 weeks of gestation. Abnormal placental thickness appears to reflect abnormal placental function; therefore, it appears to be responsible for compromised fetal and neonatal adaptations.

Hamidi et al. conducted a retrospective study on 200 term singleton pregnant mothers. Placental thickness showed a positive correlation with neonatal birth weight [18]. This corresponds with our study, in which low birth weight was associated with a thin placenta. However, in contrast with this study, we found an association between NICU admission and a thin placenta.

In our study, among 298 patients at 18-20 weeks, 36.67% of those with a thin placenta displayed FGR compared with 3.14% of those with normal placental thickness. At 30-32 weeks, 43.33% of patients with a thin placenta displayed FGR, compared with 2.34% of patients with normal placental thickness. A significant association (*P* ≤ 0.0001) was found between a thin placenta and FGR in both gestation periods under investigation. Similar results were seen in studies by Mathai et al. and Baghel et al., which showed an association between a thin placenta and FGR [19,20].

Similarly to the study done by Mathai et al., a positive correlation between placental volume and GA remained reduced in growth-restricted fetuses [19]. They found that subnormal placental thickness for a given GA may be the earliest indication of FGR. Studies have shown that diminished placental size precedes FGR, as FGR is associated with poor villous development and fetoplacental angiogenesis [21,22].

Baghel et al. found that 8 out of 10 patients with FGR were below the 10th percentile in terms of placental thickness [20]. In contrast, Miwa et al. and Verma et al. observed that placental thickness is greater in patients with FGR compared with normal placental thickness [15,23]. This contradiction could be due to a variation in sample size. More studies with large samples are required.

## Conclusions

Placental thickness can easily be evaluated by ultrasonography. Abnormal placental thickness (<10th percentile or >95th percentile) shows a significant statistical correlation with adverse fetal and neonatal outcomes as early as 18-20 weeks of gestation. Placental thickness can serve as a simple tool to aid the early prediction of adverse outcomes, and patients with abnormal placental thickness should be monitored closely. The routine measurement of placental thickness can be used as a prognostic marker for adverse outcomes in low-resource settings.
